# Hematologic Toxicity and Bone Marrow-Sparing Strategies in Chemoradiation for Locally Advanced Cervical Cancer: A Systematic Review

**DOI:** 10.3390/cancers16101842

**Published:** 2024-05-11

**Authors:** Dinah Konnerth, Aurelie Gaasch, Annemarie Zinn, Paul Rogowski, Maya Rottler, Franziska Walter, Johannes Knoth, Alina Sturdza, Jan Oelmann, Freba Grawe, Raphael Bodensohn, Claus Belka, Stefanie Corradini

**Affiliations:** 1 Department of Radiation Oncology, University Hospital, LMU Munich, 81377 Munich, Germany; 2 Department of Radiation Oncology, Comprehensive Cancer Center, Medical University of Vienna, 1090 Vienna, Austria; 3 Department of Radiation Oncology, Göttingen University Hospital, 37075 Göttingen, Germany; 4 DKFZ Hector Cancer Institute at the University Medical Center Mannheim, 69120 Heidelberg, Germany; 5 Department of Clinical Radiology and Nuclear Medicine, University Medical Center Mannheim, Medical Faculty Mannheim, Heidelberg University Mannheim, 68167 Mannheim, Germany; 6 Department of Radiation Oncology, University Hospital Tübingen, 72076 Tübingen, Germany

**Keywords:** hematologic toxicity, cervical cancer, bone marrow, chemoradiotherapy, dose constraints

## Abstract

**Simple Summary:**

Chemoradiation as a standard treatment for locally advanced cervical cancer is known to induce severe hematologic toxicity. This systematic review aims to evaluate the relationship between pelvic bone marrow irradiation and hematologic toxicity in patients undergoing platin-based chemoradiation for locally advanced cervical cancer. We seek to summarize possible dose constraints for optimal bone marrow sparing and optimize clinical strategies to mitigate treatment-related toxicities.

**Abstract:**

The standard treatment for locally advanced cervical cancer typically includes concomitant chemoradiation, a regimen known to induce severe hematologic toxicity (HT). Particularly, pelvic bone marrow dose exposure has been identified as a contributing factor to this hematologic toxicity. Chemotherapy further increases bone marrow suppression, often necessitating treatment interruptions or dose reductions. A systematic search for original articles published between 1 January 2006 and 7 January 2024 that reported on chemoradiotherapy for locally advanced cervical cancer and hematologic toxicities was conducted. Twenty-four articles comprising 1539 patients were included in the final analysis. HT of grade 2 and higher was observed across all studies and frequently exceeded 50%. When correlating active pelvic bone marrow and HT, significant correlations were found for volumes between 10 and 45 Gy and HT of grade 3 and higher. Several dose recommendations for pelvic bone and pelvic bone marrow sparing to reduce HT were established, including V10 < 90–95%, V20 < 65–86.6% and V40 < 22.8–40%. Applying dose constraints to the pelvic bone/bone marrow is a promising approach for reducing HT, and thus reliable implementation of therapy. However, prospective randomized controlled trials are needed to define precise dose constraints and optimize clinical strategies.

## 1. Introduction

Cervical cancer remains a significant global health concern, with substantial morbidity and mortality rates worldwide (an estimated 604,000 new cases and 342,000 deaths in 2020) [1,2]. Although there is a downward trend in incidence due to the HPV vaccine, particularly in industrialized nations, it remains a leading cause of cancer-related deaths among women [1,3]. Cervical cancer poses a significant health burden, necessitating effective preventive and therapeutic strategies. Human papillomavirus (HPV) infection (mainly HPV 16 and 18) stands as the primary trigger for cervical cancer development, emphasizing the importance of preventive measures such as HPV vaccination. The introduction of HPV vaccines has offered a significant reduction in the incidence of cervical cancer by targeting high-risk HPV strains [4,5].

The standard treatment for locally advanced cervical cancer typically involves a multimodal approach, consisting of external-beam radiation therapy (RT) with concomitant chemotherapy (CTx), followed by brachytherapy (BT) [6]. However, while these treatments are essential for tumor control, they might lead to treatment-related side-effects in the surrounding healthy tissues. Particularly, gastrointestinal (GI) and genitourinary (GU) toxicities, as well as hematologic toxicities such as bone marrow deficiency, may have a significant impact [7]. Prospective studies have revealed that the incidence of grade ≥ 3 HT in platin-based pelvic chemoradiation ranges between 20% and 25% [7]. The application of extended-field para-aortic radiation with increased exposure of the pelvic and lower spine bones results in even broader exposure of the overall bone marrow, consequently leading to an elevated incidence of HT [8].

Bone marrow consists of hematopoietic stem cells, from which blood cells develop through cell division and cell differentiation. In adults, blood-forming bone marrow (red or active bone marrow (ABM)) is no longer found in all bones, but only in the sternum, ribs, skull bones, clavicles, vertebrae, pelvis, scapulae and the upper ends of humeri and femora. In a 40-year-old person, the ABM in the sacrum and pelvic bones makes up around 40% of the total active bone marrow, which therefore plays a pivotal role in replenishing blood cells [9].

Understanding the nuances of bone marrow irradiation is crucial, above all, the distinction between active and inactive bone marrow compartments. A study by Robinson et al. found that active and inactive pelvic bone marrow responded differently to concomitant chemoradiation: the volume of pelvic active bone marrow (PABM) shows a median absolute decline of −0.25 g/mL compared to −0.02 g/mL for inactive pelvic bone marrow, suggesting that defining PABM is central in radiation therapy. One approach to accomplish this is using an [^18^F]FDG-PET in delineating the pelvic bone in addition to using the planning CT. ABM can be defined as having [^18^F]FDG uptake greater than the mean of the whole structure [10].

The correlation between the volume of bone marrow irradiation and dose exposure with acute HT [11,12] underscores the need for precise treatment planning and delivery techniques to minimize these adverse effects. Moreover, chemotherapy further increases bone marrow suppression, often necessitating treatment interruptions or dose reductions, which can compromise oncologic outcomes [13,14].

While advancements in radiation techniques allow for bone marrow sparing [15,16], similar progress in chemotherapeutic regimens remains limited. However, emerging targeted therapies hold promise for future improvements in treatment tolerability and efficacy [17,18].

This review article aims to consolidate the existing literature on the relationship between pelvic bone marrow irradiation and hematologic toxicity in patients undergoing primary platin-based chemoradiation for locally advanced cervical cancer. By elucidating these relationships, we seek to provide insights into bone marrow contouring methods and the optimization of clinical strategies to mitigate treatment-related toxicities.

## 2. Materials and Methods

This review was conducted in compliance with the Preferred Reporting Items for Systematic Reviews and Meta-Analyses (PRISMA) guidelines [19]. The systematic review followed the recommendations of the Preferred Reporting Items for Systematic Reviews and Meta-Analyses (PRISMA). The protocol has not been registered.

### 2.1. Search Strategy

A search of MEDLINE (via PubMed) and connected papers was conducted from 1 January 2006 to 7 January 2024. The search encompassed articles related to chemoradiation (CRT) in cervical cancer reporting on hematologic toxicities and bone marrow dose exposure. Following the removal of duplicates, two independent investigators (DK, SC) screened all records by title, abstract and full text. Any discrepancies were resolved through consensus-based discussion.

### 2.2. Search Terms

((bone marrow[Title/Abstract]) OR (hematolog*[Title/Abstract])) AND ((gynaecolog*[Title/Abstract]) OR (gynecolog*[Title/Abstract]) OR (cervical[Title/Abstract]) OR (pelvic[Title/Abstract])) AND ((cancer[Title/Abstract]) OR (carcinoma[Title/Abstract]) OR (malignan*[Title/Abstract])) AND ((radiotherapy[Title/Abstract]) OR (radiation[Title/Abstract]) OR (irradiation[Title/Abstract]) OR (chemorad*[Title/Abstract]))

### 2.3. Eligibility Criteria

The eligibility criteria for inclusion in this review article encompassed studies focusing on cervical cancer patients who underwent (chemo)radiotherapy. Specifically, we considered studies reporting on the correlation between hematologic toxicity and the radiation dose received by the pelvic bone marrow. Included studies needed to have a minimum of 10 patients and were limited to those published from 1 January 2006 to 7 January 2024. Studies with pre- or postoperative treatment settings were eligible for inclusion. The exclusion criteria comprised letters, reviews, abstracts and editorials, as well as studies published in languages other than English or German. Additionally, studies involving mixed histologies that could not be analyzed separately were excluded. The detailed eligibility criteria are presented in Table 1.

### 2.4. Data Extraction and Analysis

Data extraction was performed independently by two authors (DK, SC) using a predefined Excel sheet (Office 365, Microsoft, Redmond, WA, USA). Any discrepancies were resolved through consensus-based discussion. Analyses, where applicable, were conducted using Excel. The review examined information regarding the study population, study design, RT technique, prescribed dose to planning target volume (PTV), inclusion of patients with extended-field RT (para-aortic), chemotherapeutic regimen and number of cycles administered, bone marrow definition (methods for contouring), whether the pelvic bone was split into subvolumes, distinction between active or inactive bone marrow, use of dose constraints for the pelvic bone, compromise of other organs at risk (OARs) for bone marrow sparing, assessment of hematologic toxicity (grading score used, frequency and timing of assessment, blood components analyzed, grade of HT measured), creation of more than one RT plan, dosimetric predictors and recommended dose limitations to the pelvic bone.

For every dosimetric factor identified, we assessed the number of studies examining its link to hematologic toxicity and the proportion of those studies reporting a meaningful association. Furthermore, we outlined the threshold doses for specific dose–volume parameters that forecast hematologic toxicity.

### 2.5. Quality Assessment

The quality of the included studies was evaluated independently by two examiners (DK, SC) using the Oxford CEBM Levels 2011 criteria [20].

## 3. Results

### 3.1. Search Results

**Figure 1:**
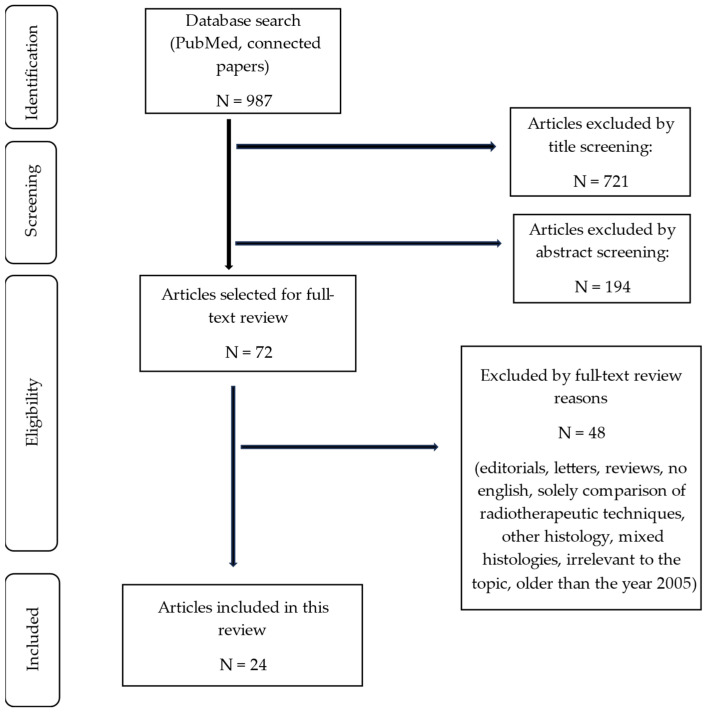
PRISMA flowchart of the screening and selection process.

Our search terms identified 987 articles extracted from the included database. The search yield and selection process are shown in Figure 1. Thereafter, we identified 24 eligible studies. All the included studies are shown in Table 2, Table 3 and Table 4. A total of 17 studies were retrospective studies, and 7 studies had a prospective study design. One study had a prospective randomized controlled trial (RCT) study design and therefore reached level 2 on the Oxford CEBM Levels of evidence; however, the majority of studies could only be assigned to level 4 on the Oxford CEBM 2011 [20].

### 3.2. Population Characteristics

The incorporated studies spanned from 2006 to 2023, involving a cumulative cohort of 1539 patients. The cohort sizes varied between 17 and 164 patients across studies. Of these, 14 studies explicitly outlined a definitive treatment regimen, wherein patients were scheduled to undergo chemoradiation followed by brachytherapy. In contrast, four studies exclusively enrolled patients who underwent chemoradiotherapy in an adjuvant setting. Additionally, five articles included patients undergoing both definitive and adjuvant treatments, while one study did not specify the treatment intention.

### 3.3. Therapy Regimens

Significant disparities exist among the studies regarding both the RT technique employed and the dosages and cycles of chemotherapy administered. Variability was also observed in terms of the PTV, encompassing differences in both dose prescription and PTV definition. Furthermore, four studies incorporated patients necessitating para-aortic irradiation, thereby inherently expanding the irradiation volume in these cohorts. Specifically, one study exclusively enrolled patients requiring para-aortic irradiation [40]. Of the included studies, 14 utilized intensity-modulated radiation therapy (IMRT) technology, with some employing IMRT techniques such as volumetric modulated arc therapy (VMAT), RapidArc or tomotherapy. Conversely, two studies exclusively employed three-dimensional (3D) treatment planning, while in seven studies, either 3D plans or IMRT plans were utilized. One study employed the anterior–posterior/posterior–anterior (AP/PA) radiation technique in 77% of patients.

The majority of patients received concomitant chemotherapy following contemporary standards [44], with cisplatin administered at 40 mg/m^2^ body surface area (BSA) in 15 out of 24 articles. In another three studies, concomitant chemotherapy also comprised cisplatin, albeit sometimes at lower doses (see Table 2). Moreover, three studies permitted the administration of carboplatin instead of cisplatin in the presence of contraindications. Induction chemotherapy was only applied in two studies: Zhang et al. [29] administered a single cycle of induction therapy (paclitaxel (175 mg/m^2^)/cisplatin (75 mg/m^2^) to 78.6% of patients, while Li et al. [12] reported 47% of patients receiving unspecified induction chemotherapy and only 15% of patients receiving concomitant chemotherapy. In the remaining 22 studies, concomitant chemotherapy was administered, with modifications to cycles under circumstances of hematologic toxicity, patients’ general condition or patient refusal.

Among the 12 articles administering chemotherapy with weekly cisplatin at 40 mg/m^2^ BSA according to contemporary standards and providing precise information on the number of cycles administered, approximately 70.5% of patients received ≥ 5 cycles of concomitant chemotherapy. The lowest value is 37.7% of patients receiving ≥ 5 cycles [30], and the highest value is 100% of patients [38].

Furthermore, variations were observed in PTV dose prescription. The lowest administered dose to the PTV was 39.6 Gy (normofractionated) [21,22,25], while the maximum dose in the PTV was reported to be 68 Gy (normofractionated) in Zhou et al. [41], although it is presumed to be a simultaneous integrated boost dose rather than a dose to the entire PTV, albeit without explicit clarification. On average, doses ranging from 45 to 50.4 Gy (normofractionated) were most commonly administered to the PTV.

### 3.4. Hematologic Toxicity

HT was assessed using either the Common Terminology Criteria for Adverse Events (CTCAE) (45%) or the RTOG criteria (55%) [45,46]. Nineteen studies performed a complete blood cell count weekly during CRT. In four articles, an additional blood cell count was conducted before the initiation of CRT [29,34,36,38], whereas in seven studies, blood values were also collected after the end of CRT, but no longer than 3 months after the end of CRT [31,38] (see Table 3). The evaluated blood components encompassed white blood cells, neutrophils, hemoglobin, thrombocytes and, in more recent investigations, lymphocytes. In instances where overall HT was not explicitly stated, Table 3 delineates the toxicities pertaining to individual blood components. It is noteworthy that hematologic toxicities of grade 2 or higher (HT 2+) were observed across all studies. HT 2+ was often found to be well over 50% in most studies that recorded this endpoint. The precise extent of HT, expressed as a percentage, along with specific information regarding the affected blood cells, if available, is documented in Table 3.

### 3.5. Pelvic Bone

#### 3.5.1. Delineation Methods

The bone marrow (BM) was delineated by outlining the pelvic bone contour on the planning CT scans. In ten studies, beyond merely considering the entire pelvic bones collectively, specific subsites were delineated to investigate potential associations between these regions and HT. Furthermore, in five articles, regions of low density within the pelvic bones were delineated and interpreted as substitutes for bone marrow. Additionally, functional imaging was utilized in seven articles alongside the planning CT scans to delineate active bone marrow. Specifically, five studies utilized [^18^F]FDG-PET-CT, while one study employed technetium-99m sulfur colloid single-photon emission tomography to quantify the standardized uptake values (SUVs). Two distinct methodologies were employed to identify active bone marrow: defining it as regions with SUVs greater than the mean SUV (SUV_mean_) of the total body or the SUV_mean_ of the entire bone. Additionally, in three studies, the pelvic bone was employed as an avoidance structure, ensuring coverage of the PTV while minimizing additional stress on other OARs.

#### 3.5.2. Correlation between Dose Received by the Bone Marrow (Pelvic Bone) and HT Grades

##### Whole Bone

Several studies have elucidated significant correlations between various volumes of irradiated whole bone and the occurrence of HT of varying grades. Notably, a significant association was observed between V5 (volume receiving 5 Gy) and HT of grade 3 or higher (HT 3+) [29]. Additionally, multiple investigations demonstrated significant correlations between V10 and a HT grade 2+, with respective studies highlighting associations with HT 3+ as well [21,22,29,41]. Moreover, seven studies emphasized a significant relationship between V20 and HT 2+, with certain studies extending this correlation to HT 3+ and HT 4+ [21,22,23,26,28,29,34,36,40]. A further analysis revealed significant correlations between V30 and HT 2+ as well as HT 3+ [28,29,36,40], while V40 exhibited significant associations predominantly with HT 2+ [24,26,28,32,35,36]. A single investigation identified a significant correlation between V45 and HT 3+ [40]. Likewise, associations between mean dose and HT 2+ or HT 3+ were highlighted in three studies [24,36,40].

##### Substructures

Two articles found a significant correlation regarding the V5 of the lower pelvic bone (LPB) and HT4+, as well as the V5 of the lumbosacral spine (LSS) and LPB-V5 and HT2+, respectively [12,34]. Similarly, three articles found a significant correlation regarding V10: LSS-V10 was found in two articles to be responsible for HT 2+ [12,35], and LPB-V10 was found to be significantly correlated with HT 2+ in [12,30]. Regarding V20, significant correlations were found in one study with LSS-V20 and in another one with LPB-V20 for HT 2+ [12]. In one case, LPB-V20 was correlated with HT 4+ [34], and in one case, V20-Ilium-PB was correlated with HT 2+ [33]. V40 was additionally investigated in three articles. LSS-V40, relative-LPB-V40 (relative dose volume of BM irradiation of LPB), LSS-V40 and LPB-V40 were associated with HT 2+ [12,28,30]. The Dmean of substructures was also investigated in some of the included studies. Two studies showed a significant correlation between LSS-Dmean and HT 2+ [35], as well as the iliac crest Dmean and HT 4+ [34]. Some other significant relationships were reported, including V15, V25, V30 and V50 (see Table 3, Table 4 and Table 5).

##### Correlation between Active Bone Marrow and HT

Three articles found a correlation between the V10 of pelvic ABM (PABM) and HT 3+ [37,40,41]. Two articles found a correlation between PABM-V20 and HT 3+ [40,41], and another two articles found a significant correlation between PABM-V30 and HT 3+ [40,42]. A further three articles discovered a significant correlation between PABM-V40 and HT 3+ [39,41,42]. Notably, one article highlighted a significant correlation between PABM-V45 and HT 3+ [40]. Moreover, two articles revealed a significant correlation between PABM-Dmean and HT 3+ [37,40], while another two articles demonstrated a significant correlation between PABM volume and HT 3+. Wang et al. [42] identified that a baseline PABM volume exceeding 387.5 cc significantly reduces the probability of HT 3+. Conversely, Khullar et al. [39] highlighted that a baseline PABM volume of ≥1201 cc is necessary to mitigate the risk of HT 3+. Remarkably, lower volumes of PABM at baseline were highly predictive of HT 3+.

##### Low-Density Bone Marrow Spaces

Despite their prior inclusion in the preceding results section, the findings for low-density bone marrow spaces are summarized as follows: Five articles defined BM as the regions of low density within the respective osseous structures [32,33,34,35,36]. Among these investigations, two articles failed to establish significant correlations upon multivariate analysis [33,34]. Mahantshetty et al. [32] discerned that a volumetric threshold of pelvic bone marrow V40 < 40% was notably linked with HT 2+. Similarly, Huang et al. [35] highlighted correlations, apart from V40, between lumbosacral spine marrow LSS-V10, V20 and V40, as well as Dmean, and HT 2+. Furthermore, Singareddy et al. [36] proposed the following dose constraints for the mitigation of adverse effects: whole pelvic bone marrow V20, V30 and V40 should be maintained below thresholds of 71.75%, 49.75% and 22.85%, respectively, with Dmean not exceeding 28.85 Gy.

##### Recommended Cut-off Values

The recommended cut-off values for mitigating the incidence of HT 2+ vary across different volumes of irradiated whole bone. Notably, the recommended thresholds include V10 < 90–95%, V20 < 65–86.6% and V40 < 22.8–40% (see Table 5). Additionally, functional imaging studies identified slightly adjusted cut-off values for active bone marrow volumes: V10 < 95.5%, V20 < 80.5% and V40 < 23.5%. Recommendations for sparing volumes of the pelvic bone and ABM are also provided, with pelvic bone V10 ≥ 230 cc and pelvic active bone marrow: V10 ≥ 179 cc, V20 ≥ 186 cc, V40 ≥ 738 cc proposed to minimize the risk of HT 2+ [41]. An overview of the recommended dose constraints is comprehensively detailed in Table 5.

In the included studies, various dose and/or volume parameters were examined to determine their correlation with hematologic toxicity. While several significant associations were identified, not every parameter currently has a recommended constraint established.

## 4. Discussion

The standard treatments for locally advanced cervical cancer, including pelvic chemoradiation, pose risks of hematologic toxicities due to bone marrow dose exposure. Specifically, the irradiation of bone marrow in the pelvic region has been recognized as a significant cause of hematologic toxicity, leading to efforts to reduce its effects. This is particularly important to ensure that the administration of simultaneous chemotherapy is not jeopardized.

One strategy involves implementing pelvic bone marrow dose constraints during the planning phase of radiotherapy with the aim of reducing the incidence and severity of HT during treatment.

An interruption of therapy and, thus, an extension of the overall treatment time has a negative impact on the oncological outcome and should always be avoided [47]. For example, it was shown that stage III disease accounted for most adverse effects from the prolongation of OTT. A significant increase in the relative risk of local recurrence and death was found when the OTT was >52 days compared with a shorter duration [48].

This review article offers an analysis of research articles investigating the relationship between bone marrow dose exposure in patients with locally advanced cervical cancer and the occurrence of HT. In total, 24 articles were examined, revealing dosimetric predictors and recommending constraints to avoid/reduce the incidence of HT using three distinct methods for delineating bone marrow: whole pelvic bone, lower-density marrow spaces and functional imaging-based active bone marrow contouring.

Hematologic toxicity:

The impact of the standard chemoradiotherapy regimen [44] for cervical carcinoma on the immune system shows substantial perturbations within the active bone marrow compartment [49]. Elicin et al. emphasize the enduring impairment persisting post-treatment even after a three-month period [38], with some extent of marrow recovery noted only after a year-long interval [50]. Endpoint analyses across the reviewed studies predominantly focused on HT grades 2+ or 3+, revealing a notable incidence exceeding 30% and 40%, respectively. However, the recommended dosimetric cut-off values were largely similar for HT 2+ and HT 3+. This can probably also be explained by the very unequal study settings: In some studies, chemotherapy was not administered if a certain degree of depletion of blood cells was seen [23,35] in order to avoid the progression of depletion. Indeed, the development of HT is influenced not only by RT but also by CTx interventions [51]. However, discerning the precise contribution of each therapeutic modality, namely RT and CTx, to HT remains elusive.

Li et al. observed a significant increase in HT incidence when comparing concomitant CTx to RT alone (70.21% vs. 29.79%; *p* = 0.001). However, their study also permitted induction chemotherapy, revealing that patients subjected to this additional regimen experienced higher HT rates (63.83% vs. 36.17%; *p* = 0.01) [12]. This disparity likely arises from the escalated dosage administered during induction CTx relative to concomitant CTx. Despite this variation, we opted to include both Li et al.’s study and that of Zhang et al. [29] in our review, notwithstanding the administration of induction CTx. Regardless, the chemotherapy protocols across the included studies were markedly heterogeneous (notably permitting carboplatin in three studies), with carboplatin being recognized for its more pronounced hematotoxicity. [52,53]. Additionally, the diversity in the number of cycles administered across the studies, as well as the sparing documentation of the number of CTx cycles administered, further underlines the complexity of this comparison (see Table 2).

In contrast, Rose et al. reported a lack of significant correlation between dosimetric bone marrow parameters and the probability of having one or more CTx cycles held [22].

Chen et al.’s investigation elucidated that the effect of bone marrow irradiation on the nadir of neutrophils was masked by CTx when more than four cycles of concomitant CTx were administered. Additionally, Chen identified that the dose received by the ilium exerted a more pronounced influence on neutrophil decline, whereas the irradiation dose to the lower pelvis had a greater impact on hemoglobin, platelets and the neutrophil-to-lymphocyte ratio (NLR). Moreover, high-dose irradiation predominantly affected hemoglobin and platelet levels, whereas NLR was more significantly influenced by low-dose irradiation. Neutrophil counts were susceptible to both low- and high-dose irradiation [30].

Generally, the existing literature indicates that lymphocytes are most sensitive to irradiation [54,55]. In four of the studies encompassed within this review, lymphocyte levels were assessed [29,30,34,43], revealing a significant reduction in lymphocyte counts (see Table 3). Thus, the studies examined in this review demonstrated a wide array of observations and experiences regarding the influence of the therapy on the individual blood components and regarding the interactions with chemotherapy.

Bone marrow contouring:

Studies have already shown that bone marrow reacts very sensitively to radiation, although there are differences between the various progenitor cells. Notably, studies have elucidated the increased radiosensitivity of neutrophils and select lymphocytic subsets [56], with even minimal radiation dose exposure precipitating both acute and long-term bone marrow-related side effects [57,58].

Given that roughly 50% of active bone marrow is localized within the pelvic bones, encompassing the proximal femur and lumbosacral spine [59,60], it can be assumed that pelvic irradiation has an effect on hematopoietic stem cells, stromal components and microcirculatory networks. Although bone marrow has historically been ignored as an OAR in radiation planning, robust evidence suggests its vulnerability, particularly in cohorts necessitating extended-field radiotherapy, wherein the lumbosacral bone marrow may be substantially implicated [50,61]. Only one of the included studies (Yan et al. [40]) exclusively examined patients subjected to para-aortic irradiation; in some of the remaining studies, patients with an indication for para-aortic irradiation were included but not analyzed separately (see Table 4). Yan et al.’s dosimetric predictors show agreement within the spectrum of the other recommended dose constraints and were in the medium range. Consequently, there is no conclusive evidence to suggest that HT would have significantly exceeded the values observed in the other studies analyzed.

It is essential to delineate the bone marrow, particularly its active regions, during radiation planning, and subsequently implement protective measures to spare its volume. One viable approach involves the integration of functional imaging modalities such as [^18^F]FDG-PET alongside radiation planning to discern active bone marrow regions [62]. The methodologies employed across the reviewed studies to identify ABM encompassed criteria such as ABM ≥ SUV_mean_-WB, ≥SUV_mean_-WPB and ≥SUV_mean_-WPBM.

An alternative method employed in select studies involved employing “freehand contouring” techniques to delineate lower-density marrow regions on planning CT scans, utilizing these areas as proxies for ABM. Notably, Mahantshetty et al. [32] demonstrated that using bone cavities as surrogates for BM allowed for the identification of dose parameters significantly correlated with HT 2+ (V40 < 40%). Conversely, in studies conducted by Kumar and Lewis, multivariate analyses failed to yield significant correlations for dose–volume histogram parameters pertaining to contoured low-density regions [33,34]. Consequently, it is recommended that functional imaging techniques be utilized for the accurate identification of ABM. In the absence of functional imaging resources, employing the entire bone as a surrogate may be sufficient.

Rose et al. further investigated the potential association between dose parameters (V10, V20, V30, V40) reaching the inactive yellow bone marrow and HT. As anticipated, their findings revealed no discernible correlation [37]. This outcome aligns with the established understanding that the yellow bone marrow houses mesenchymal stem cells crucial for cartilage, bone and adipose tissue generation [63].

Radiation technique:

The radiation modality employed can influence both the extent of BM irradiation and the resultant dose exposure. IMRT offers superior conformality, facilitating the sparing of adjacent OARs [64]. While extensively studied for organs at risk, such as the bladder and bowel [65], this principle extends to pelvic bone sparing when compared to conventional techniques like four-field box irradiation [66], AP/PA irradiation [16] or 3D irradiation [67].

However, despite evidence suggesting IMRT’s efficacy in pelvic bone sparing, the translation of this reduction in radiation exposure to a significant decrease in HT is not consistently evident. Nonetheless, investigations comparing bone marrow-sparing IMRT (BMS-IMRT) with conventional IMRT consistently demonstrate fewer adverse effects regarding HT when BM considerations are taken into account [68,69].

Consequently, delineating the pelvic bone as an OAR and minimizing its irradiation should be standard practice, provided that it does not compromise PTV coverage and does not subject other OARs to undue stress [70]. A recently published study has even shown that it is extremely useful to define ABM. Wang et al.’s prospective clinical trial demonstrated that ABMS VMAT significantly reduced grade 3+ HT and improved chemotherapy delivery in locally advanced cervical cancer patients undergoing chemoradiotherapy [71].

Among the studies encompassed within our analysis, 3D and IMRT techniques were predominantly used. The studies that used different irradiation techniques in their collective showed contradictory results. For instance, Yan et al. reported no statistically significant disparity in the risk of developing HT 3+ across irradiation techniques (3D or IMRT) [40]. In contrast, Ajayakumar et al., who also employed either 3D or IMRT technique, presented conflicting findings [28]. They demonstrated markedly reduced HT within the IMRT cohort.

Despite these inconsistencies, the literature predominantly shows the advantages of the IMRT technique. Thus, IMRT emerges as the preferred irradiation modality whenever feasible.

Dosimetric parameters:

The dosimetric parameters associated with HT were identified. Most authors considered the entire pelvic bone as a surrogate for bone marrow. The recommended threshold values encompass V10 < 90–95%, V20 < 65–86.6% and V40 < 22.85–40%. The notable breadth of these ranges may be attributed to substantial variations in study design, which include differences in RT techniques, dose prescriptions, chemotherapy regimens and the numbers of cycles applied, as well as the timing of HT assessments. Such multidimensional factors exert discernible influences on HT outcomes, such that different results were to be expected. Some studies investigated specific subsites within the pelvic bone for nuanced exploration.

For instance, the lumbosacral spine harbors approximately 25% of active bone marrow, while the proximal femora contain about 10%, and the pelvic bones, including the os ischium, os ilium and os pubis, account for roughly 30% [60]. The dosimetric parameters pertaining to these substructures demonstrated comparable associations with HT. Notably, significant correlations were observed for two parameters within each of the LPB, LSS and os ilium: LPB-V5 ≤ 95%, LPB-V20 ≤ 45%, LSS-V10 < 87%, LSS-V40 < 50.9%, ilium-PB-V20 ≤ 90% and Dmean iliac crest ≤ 31 Gy. These findings suggest that all subsites must be spared equally from radiation exposure.

Studies utilizing functional imaging to assess active bone marrow also evidenced significant correlations between certain dose parameters (V10, V20, V30, V40, V45) and HT. Nevertheless, the recommended doses do not differ significantly from those given for the whole pelvic bone. This may be due to the fact that the regions with active bone marrow are naturally also included in the delineated whole pelvic bone. However, in scenarios where sparing the entire pelvic bone proves unfeasible due to anatomical constraints, the precise localization of ABM regions becomes paramount.

Various studies have identified different relevant dose parameters associated with HT. Specifically, while some investigations have found associations within the V10 and V20 ranges, i.e., in the low-dose spectrum, others have exclusively revealed correlations within higher dose ranges, such as V40 (see Table 4 and Table 5). One plausible explanation for this divergence could be multicollinearity, a phenomenon that yields wider confidence intervals and subsequently diminishes the reliability of probabilities regarding the impact of independent variables within a model. Another reason is certainly that not all dose levels were investigated in all studies.

Additionally, certain studies opted for volume constraints over dose constraints and considered bone marrow as a parallel organ. Indeed, the manifestation of HT likely arises from the interplay of both the dose and volume reaching the irradiated bone marrow. Ultimately, the discordant findings regarding dosimetric parameters correlated with HT highlight the complexity of this relationship, mandating the need for further comprehensive investigations.

Further research is needed to determine the optimal dose and volume constraints for bone marrow, an essential organ at risk, especially in the context of locally advanced cervical carcinoma. The current literature lacks prospective studies and long-term data, with only two studies examining post-treatment blood assessments three months after the end of treatment [31,38]. Prolonged bone marrow impairment can hinder subsequent therapies, notably in the context of ongoing research exploring adjuvant immunotherapy [72]. In conclusion, this review highlights the complex relationship between bone marrow irradiation and hematologic toxicity in locally advanced cervical cancer patients, emphasizing the need for continued research to improve treatment outcomes.

## 5. Conclusions

In conclusion, our systematic review emphasizes the significant impact of hematologic toxicity in locally advanced cervical cancer patients undergoing chemoradiotherapy, with HT of grade 2 and higher observed in over 50% of cases across all studies. The correlations between active pelvic bone marrow dose exposure and severe HT highlight the importance of dose optimization strategies. While dose recommendations for pelvic bone and bone marrow sparing show promise in reducing HT, further research, particularly through prospective randomized controlled trials, is crucial to establish precise dose constraints and optimize clinical approaches for improving treatment outcomes in this patient population.

## Figures and Tables

**Table 1:** Eligibility criteria.

Inclusion Criteria	Exclusion Criteria
Patients with cervical cancer who received (chemo)radiation therapy	Letters, reviews, abstracts and editorials
Studies reporting on the correlation between hematologic toxicity and the radiation dose received by the pelvic bone marrow	Studies published in languages other than English or German
Studies with a minimum of 10 patients	Mixed histologies that cannot be analyzed separately
Publications from 2006 onwards	Studies before the year 2006
Both pre- and postoperative settings considered	

**Table 2:** Included studies—general information on study design, radiotherapy (RT) and chemotherapy (CTx) details.

Author and Year	Number of Patients	Study Design	RT Intention	Dose Prescription	RT Technique	Chemotherapeutic Regimen	CTx Cycles (Patients in %)
Mell, 2006 [21]	37	Retrospective case series	Definitive (91.9%)/postoperative (8.1%)	39.6–50.4 Gy	IMRT + BT	Cisplatin 40 mg/m^2^ weekly	6 cy: 2.7%, 5 cy: ≈46%, 4 cy: ≈35%
Rose, 2011 [22]	81	Retrospective case series and validation cohort	definitive	39.6–50.4 Gy	IMRT + BT	Cisplatin 40 mg/m^2^ weekly	4 cy: 20%, 5 cy: 34%, 6 cy: 25%, 55% at least 1 cy
Albuquerque, 2011 [23]	40	Retrospective case series	Definitive	45 Gy	3D	Cisplatin 40 mg/m^2^ weekly	Not mentioned
Klopp, 2013 [24]	40	Prospective cohort study	Postoperative	50.4 Gy	IMRT	Cisplatin 40 mg/m^2^ weekly	5 cy ≥ 83%, 4 cy ≥ 90%, 4 cy < 7.5%
Zhu, 2015 [25]	102	Retrospective cohort study	Postoperative/definitive	39.6–50.4 Gy	IMRT (97.1%)/3D (2.9%) +BT	Cisplatin 40 mg/m^2^ weekly	6 cy: 20%, 5 cy: 38%, 4 cy: 22%, 3 cy: 11%, 2 cy: 5%, 1 cy: 4%
Chang, 2016 [26]	100	Retrospective case series/cohort study	Definitive	50.4–56 Gy	3D (32%)/IMRT (37%)/ IMRT (RapidArc; 31%) + BT	Cisplatin 25 mg/m^2^	3–6 cy
Li, 2016 [12]	100	Retrospective case series	Postoperative	40–50 Gy	2D (AP/PA: 77%)/IMRT (23%)	52% received CT (paclitaxel/nedaplatin). Concomitantly: 15%; induction CT: 47%.	6 cy ≥ 4%, 5 cy: 1%, 4 cy: 5%, 3 cy: 4%, 2 cy: 17%, 1 cy: 21%
Bosque, 2018 [27]	59	Retrospective cohort study	Not mentioned	45–50.4 Gy	3D + BT	Cisplatin (93.2%)/carboplatin (6.7%) weekly	5 cy ≥ 22%, 5 cy < 77.9%
Ajayakumar, 2018 [28]	47	Prospective observational study	Definitive	45–50.4 Gy	3D (53.2%)/IMRT (46.8%) + BT	Cisplatin 40 mg/m^2^ weekly	Not mentioned
Zhang, 2023 [29]	117	Retrospective case series	Definitive	50.4 Gy	IMRT (24.79%)/VMAT (75.21%) + BT	Induction CT: (paclitaxel/cisplatin; 78.63%). Concomitant CT: cisplatin 25 mg/m^2^ weekly	5 cy: 43.6%, 3–4 cy: 56.4%
Chen, 2023 [30]	69	Retrospective case series	Definitive (36.2%)/postoperative (63.8%)	45–50.4 Gy	IMRT +BT	Cisplatin 40 mg/m^2^ weekly	3–4 cy: 62.3%, 5–6 cy: 37.7%
Sun, 2023 [31]	40	Prospective observational study	Postoperative	50 Gy	IMRT (50%) and PBMS-IMRT (50%)	Cisplatin 35–40 mg/m^2^ weekly	Not mentioned
Mahantshetty, 2012 [32]	47	Retrospective case series	Definitive	50 Gy	IMRT + BT	Cisplatin 40 mg/m^2^ weekly	4 cy ≥ 95%, 2 cy ≤ 5%
Lewis, 2018 [33]	75	Retrospective case series	Postoperative	50 Gy	IMRT (TOMO)	Cisplatin 40 mg/m^2^ (98.7%)/carboplatin (1.3%) weekly	6 cy: 1.3%, 5 cy: 69.3%, 4 cy: 24%, 3 cy: 1.3%, 2 cy: 2.7%, 1 cy: 1.3%
Kumar, 2019 [34]	114	Retrospective case series	Definitive	45 Gy (+Boost up to 60 Gy)	3D (75.4%)/IMRT (24.6%) + BT	Cisplatin 40 mg/m^2^ (89.5%) or carboplatin AUC2 (10.5%) weekly in case of renal impairment	5 cy: 76.3%, 4 cy: 19.3%, 4 cy < 4.4%
Huang, 2020 [35]	164	Prospective RCT	Definitive	50.4 Gy	IMRT + BT	Cisplatin 40 mg/m^2^ weekly	PBMS group: 6 cy: 48.8%, 5 cy: 40.2%, 4 cy: 8.5%, 2 cy: 1.2%, 1 cy: 1.2% Control group: 6 cy: 45.1%, 5 cy: 37.8%, 4 cy: 17.1%
Singareddy, 2021 [36]	34	Prospective observational study	Definitive	50 Gy	IMRT (VMAT) + BT	Cisplatin 40 mg/m^2^ weekly	5 cy: 85.2%, 4 cy: 11.8%, 3 cy: 2.9%
Rose, 2012 [37]	26	Retrospective case series	Definitive 81%; postoperative 19%	45–50.4 Gy (+ Boost up to 60–66 Gy)	IMRT + BT	Cisplatin 40 mg/m^2^ weekly	6 cy: 27%, 5 cy: 35%, 4 cy: 15%, 3 cy: 15%, 1 cy: 8%
Elicin, 2014 [38]	17	Retrospective case series	Definitive	45–50.4 Gy (+ Boost up to 60 Gy)	IMRT (TOMO) + BT	Cisplatin 40 mg/m^2^ weekly	5 cy: 100%
Khullar, 2017 [39]	21	Retrospective case series	Definitive	45–50.4 Gy	3D (76%)/IMRT (14%)/both (9%)	Cisplatin	2–7 cy; ≥5 cy: 61.9%
Yan, 2018 [40]	38	Retrospective case series	Definitive	45 Gy (+ Boost up to 55–60 Gy)	3D/IMRT + BT	Cisplatin 40 mg/m^2^ weekly	Not mentioned
Zhou, 2018 [41]	31	Retrospective case series	Definitive/postoperative	45–68 Gy	3D (58%)/IMRT (42%)	Cisplatin 40 mg/m^2^ weekly	0 cy: 29%, 1–3 cy: 19%, 4–5 cy: 32%, 6–7 cy: 19%
Wang, 2019 [42]	39	Prospective cohort study	Definitive	45 Gy (+ Boost 10–20 Gy)	IMRT (VMAT) + BT	Cisplatin 30–40 mg/m^2^ weekly	5 cy: 84.6%, 4 cy: 15.4%
Williamson, 2022 [43]	101	Prospective	Definitive	45–50.4 Gy (+ Boost up to 59.4 Gy)	IMRT + BT	Cisplatin 40 mg/m^2^ weekly	≥5 cy > 80%

Abbreviations: RT: radiotherapy; CTx: chemotherapy; Gy: Gray; RCT: randomized controlled trial; 3D: three-dimensional conformal radiotherapy; BT: intracavitary and/or interstitial brachytherapy; IMRT: intensity-modulated radiotherapy; AP/PA: anterior–posterior/posterior–anterior; VMAT: volumetric modulated arc therapy; PBMS: pelvic bone marrow sparing; TOMO: tomotherapy; cy: cycles.

**Table 3:** Included studies—information regarding hematologic toxicity.

Author and Year	HT Assessment Method	HT Assessment Frequency	Blood Components Analyzed	HT Incidence
Mell, 2006 [21]	RTOG	Weekly during CRT	WBC, ANC, Hb, PLT	G2+ LKP 43.2%, NTP 18.9%
Rose, 2011 [22]	RTOG	Weekly during CRT	WBC, ANC, Hb, PLT	G2+ LKP 74%, NTP 48%
Albuquerque, 2011 [23]	CTCAE 3.0	During CRT	WBC, ANC, Hb, PLT	G2+ HT 67.5%
Klopp, 2013 [24]	CTCAE 3.0	Not mentioned	Not mentioned	G2+ HT 58%, G3+ HT 25%
Zhu, 2015 [25]	Not mentioned	Weekly during CRT	WBC, ANC, Hb, PLT	Not mentioned
Chang, 2016 [26]	Not mentioned	Weekly during CRT	WBC, ANC, Hb, PLT	G2+ LKP: 3D/IMRT/RapidARC: 100%/78.4%/80.6% G3+ LKP: 78.1%/40.5%/48.4% G2+ NTP: 93.7%/64.9%/67.7% G3+ NTP: 65.6%/27.0%/29.0%
Li, 2016 [12]	RTOG	Weekly and 120 days after start of RT	WBC, ANC, Hb, PLT	G2+ HT 47%
Bosque, 2018 [27]	RTOG	Not mentioned	WBC, ANC, Hb, PLT	G2+ HT 50.8%
Ajayakumar, 2018 [28]	RTOG	Weekly during CRT	Not mentioned	G2+ 38.3% (3D: 72.2%/IMRT: 27.8%)
Zhang, 2023 [29]	CTCAE 4.0	Baseline, weekly during CRT and at 1 mo FU	LYM	G3+ LYP 68.38%
Chen, 2023 [30]	RTOG	Weekly during CRT	LYM, ANC, Hb, PLT	G2+ NTP: 50.7%, Anemia: 21.7%, Thrombocytopenia: 24.6%.
Sun, 2023 [31]	Not mentioned	Until 3-month FU	Not mentioned	IMRT: G2+ 45% vs. PBMS-IMRT:G2+ 25%. (*p* = 0.038).
Mahantshetty, 2012 [32]	RTOG	Weekly during CRT	WBC, ANC, Hb, PLT	G2+ LKP 53%, NTP 29.8%
Lewis, 2018 [33]	CTCAE 3.0	Weekly during CRT	WBC, ANC, Hb, PLT	G2+ 57.4%, G3+ HT 14.7%
Kumar, 2019 [34]	CTCAE 4.0	Baseline, weekly during CRT and at least once within 2 weeks FU	WBC, LYM, ANC, Hb, PLT	G4+ LKP 2.6%, LYM 12.3%
Huang, 2020 [35]	RTOG	Weekly during CRT	WBC, ANC, Hb, PLT	G2+ HT 50%
Singareddy, 2021 [36]	RTOG	Baseline and weekly during CRT	WBC, Hb, PLT	G2+ HT 50%
Rose, 2012 [37]	RTOG	Weekly during CRT	WBC, ANC, Hb, PLT	G3+ LKP 38.5%, NTP 23.1%
Elicin, 2014 [38]	RTOG	1 week before and weekly during CRT 3 months after CRT, and at last FU	WBC, ANC, Hb, PLT	G3+ LKP 35%, NTP 35%
Khullar, 2017 [39]	CTCAE 4.0	Weekly to 6 weeks after end of CRT	Not mentioned	G3+ HT 71.4%
Yan, 2018 [40]	CTCAE 4.0	Weekly during RCT	WBC, ANC, Hb, PLT	G3+ HT 50%
Zhou, 2018 [41]	CTCAE 4.0	Weekly and one week after CRT	WBC, ANC, Hb, PLT	G3+ HT 77%
Wang, 2019 [42]	CTCAE 3.0	Weekly to two weeks after CRT	WBC, ANC, Hb, PLT	G3+ LKP 46.2%, NTP 2.5%
Williamson, 2022 [43]	Not mentioned	Not mentioned	LYM, ANC, Hb, PLT	PET-BMS-IMRT vs. IMRT: significantly reduced G3+ neutropenia (13% vs. 35%)

Abbreviations: HT: hematologic toxicity; CTCAE: common terminology criteria for adverse events; RTOG: radiation therapy oncology group; CRT: chemoradiotherapy; FU: follow-up; WBC: white blood cells; ANC: absolute neutrophil count; Hb: hemoglobin; PLT: platelets (thrombocytes); LYM: lymphocytes; LKP: leukopenia; NTP: neutropenia; LYP: lymphopenia; BMS: bone marrow sparing.

**Table 4:** Included studies: information regarding bone marrow sparing.

Author and Year	Bone Marrow Delineation Method	Bone Marrow Substructure Definition	Bone Marrow Dose Constraint during Treatment Planning	Distinction between ABM and IBM	Extended-Field RT (Para-Aortic)	Dosimetric Predictors
Mell, 2006 [21]	CT-based PB contour	yes (LSS, IL, LPB)	no	no	no	PB-V10, V20
Rose, 2011 [22]	CT-based PB contour	no	no	no	no	PB V10, V20
Albuquerque, 2011 [23]	CT-based PB contour	yes (LSS, IL, LPB, PB+IL+LPB, WPB+LSS)	no	no	no	PB-V20
Klopp, 2013 [24]	CT-based PB contour	no	no	no	no	PB-V40, Dmean
Zhu, 2015 [25]	CT-based PB contour	Yes (LSS, IC, LPB)	individual	no	no	PB-Dmean, V20, V30, V40, LSS-V10, V40, LPB-V20, V30
Chang, 2016 [26]	CT-based PB contour	no	no	no	no	PB-V20, V40
Li, 2016 [12]	CT-based PB contour	Yes (WPB, LSS))	no	no	no	LSS-V5-40 and LPB-V5-40
Bosque, 2018 [27]	CT-based PB contour	no	no	no	not mentioned	none
Ajayakumar, 2018 [28]	CT-based PB contour	Yes (WPB, LSS)	yes	no	no	PBM: V20, 30, 40. LSSBM: V40
Zhang, 2023 [29]	CT-based PB contour	no	no	no	not mentioned	PB V5, V10, V20 and V30
Chen, 2023 [30]	CT-based PB contour	Yes (IL, LPB, LSS)	no	no	no	Hb: R(elative)-LPB-V10, R-LPB-V25, R-LPB-V50, R-LPB-mean, A(bsolute)-LPB-V15, A-LPB-V25 and A-LPB-V30. PLT: R-LPB-V40. ANC: R-IL-V15 and R-IL-V50 and A-LPB-V50. NLR: R-LPB-V15 and A-PBM-mean
Sun, 2023 [31]	CT-based PB contour	no	yes (V20 < 76% and V40 < 35% in the PBMS group)	no	no	none
Mahantshetty, 2012 [32]	CT-based PB contour + BM defined as the low-density regions within the corresponding bones	yes (SB, IL, IS, LPB, LSS, WPB)	no	no	not mentioned	PBM-V40
Lewis, 2018 [33]	CT-based PB contour + BM defined as the low-density regions within the corresponding bones	yes (WPB+LS, LS, SB, IL, IS, FB, WPB, LPB)	no	no	not mentioned	Ilium-PB V20
Kumar, 2019 [34]	CT-based PB contour + BM defined as the low-density regions within the corresponding bones	yes (LSS, IC, LPB)	no	no	yes (13.2%)	PB-V20, LPB-V5,20, Iliac crest-Dmean
Huang, 2020 [35]	CT-based PB contour + BM defined as the low-density regions within the corresponding bones	yes (WPB, LSS)	yes	no	no	PB: PB-V40, LSS-V10, Dmean; PBM: PBM-V40, LSSBM -V10, V20, V40, Dmean
Singareddy, 2021 [36]	CT-based PB contour + BM defined as the low-density regions within the corresponding bones	no	no	no	no	WPBM-V20,30,40 and Dmean
Rose, 2012 [37]	CT-based PB contour, [^18^F]FDG-PET based ABM (≥SUVmean WPB)	no	no	yes	yes (23%)	PABM Dmean
Elicin, 2014 [38]	CT-based PB contour, [^18^F]FDG-PET based ABM (≥SUVmean WPB)	no	no	yes	yes (23.5%)	none
Khullar, 2017 [39]	CT-based PB contour, [^18^F]FDG-PET based ABM (≥SUVmean WB)	no	no	yes	not mentioned	PABM volume, PABM-V40
Yan, 2018 [40]	CT-based PB contour, [^18^F]FDG-PET based ABM (≥SUVmean WPB)	no	no	yes	yes (100%)	PB-V20, V30, V45, Dmean; PABM-V10,20,30,45, Dmean
Zhou, 2018 [41]	CT-based PB contour, [^18^F]FDG-PET ABM (≥SUVmean WB)	no	no	yes	not mentioned	PB-V10; PABM V10, 20,40 Gy
Wang, 2019 [42]	CT-based PB contour, Tc-99m SPET (≥SUVmean WB)	no	no	yes	no	PABM volume, PABM-V30, V40
Williamson, 2022 [43]	CT-based PB contour, [^18^F]FDG-PET-based ABM (≥SUVmean WPBM)	no	yes (PBM and ABM mean doses were constrained to <27 Gy and <28.5 Gy; V10 <90% and V20 <75%)	yes	no	none

Abbreviations: ABM: active bone marrow; IBM: inactive bone marrow; CT: computed tomography; PB: pelvic bone; BM: bone marrow; LSS: lumbosacral spine; IL: os ilium; LPB: lower pelvic bone; SB, sacral bone; LS: lumbar spine; WPB: whole pelvic bone; IC: iliac crest; IS: os ischium; FB: femoral bone; Vx: volume receiving x Gy; [18F]FDG-PET: 2-deoxy-2-[18F]fluoro-D-glucose positron emission tomography; Tc-99m SPET: technetium-99m sulfur colloid single-photon emission tomography; WB: whole body; SUV: standardized uptake value; WPBM: whole pelvic bone marrow; PBM: pelvic bone marrow; LSSBM: lumbosacral spine bone marrow; PBMS: pelvic bone marrow sparing; Hb: hemoglobin; PLT: platelets (thrombocytes); ANC: absolute neutrophil count; NLR: neutrophil-to-lymphocyte ratio; PABM: pelvic active bone marrow.

**Table 5:** Recommended dose constraints from the included studies.

Parameter	Recommended Dose Constraints
**Whole Pelvic Bone (Marrow)**	
V10	<90%, <95%
V20	≤65%, <71.7%, <76%, <78.6%, <79.4%, <86.6%
V30	<47.1%, <49.7%, <57%
V40	<22.8%, <28%, <29%, ≤37%, <40%
V45	< 20.4%
Dmean	<28.8 Gy, <30.3 Gy, ≤34.2 Gy, <39.0 Gy,
Volume spared 10 Gy	≥230 cc
**Substructures (Bone Marrow)**	
LPB-V5	≤95%
LSS-V10	<87%
LPB-V20, IL-PB-V20	≤45%, ≤90%
LSS-V40	<50.9%
Dmean IC	≤31 Gy
**Pelvic Active Bone Marrow**	
V10	<95.5%
V20	<80.5%
V30	<46.5%, <59.6%
V40	<23.5%
V45	<31.7%
Dmean	<26.8 Gy, <32.4 Gy
Baseline PABM volume	>387.5 cc, ≥1201 cc
Volume spared (V10, V20, V40)	≥179 cc, ≥186 cc, ≥738 cc

Abbreviations: LPB: lower pelvic bone; LSS: lumbosacral spine; IL: os ilium; IC: iliac crest; PABM: pelvic active bone marrow.

## Abbreviations

3D:	three-dimensional conformal radiotherapy
[^18^F]FDG-PET:	2-deoxy-2-[18F]fluoro-D-glucose positron emission tomography
ABM:	active bone marrow
ANC:	absolute neutrophil count
AP/PA:	anterior–posterior/posterior–anterior
BM:	bone marrow
BMS:	bone marrow sparing
BSA:	body surface area
BT:	brachytherapy
CRT:	chemoradiotherapy
CT:	computed tomography
CTCAE:	Common Terminology Criteria for Adverse Events
CTx:	chemotherapy
Cy:	cycles
FB:	femoral bone
FU:	follow-up
GI:	gastrointestinal
GU:	genitourinary
Gy:	Gray
Hb:	hemoglobin
HPV:	human papillomavirus
HT:	hematologic toxicity
IBM:	inactive bone marrow
IC:	iliac crest
IL:	os ilium
IMRT:	intensity-modulated radiation therapy
IS:	os ischium
LKP:	leukopenia
LPB:	lower pelvic bone
LPBM:	lower pelvic bone marrow
LS:	lumbar spine
LSS:	lumbosacral spine
LSSBM:	lumbosacral spine marrow
LYM:	lymphocytes
LYP:	lymphopenia
NLR:	neutrophil-to-lymphocyte ratio
NTP:	neutropenia
OAR:	organ at risk
PABM:	pelvic active bone marrow
PB:	pelvic bone
PBM:	pelvic bone marrow
PBMS:	pelvic bone marrow sparing
PLT:	platelets (thrombocytes)
PTV:	planning target volume
RCT:	randomized controlled trial
RT:	radiation therapy
SB:	sacral bone
SUV:	standardized uptake value
Tc-99m SPET:	technetium-99m sulfur colloid single-photon emission tomography
TOMO:	tomotherapy
VMAT:	volumetric modulated arc therapy
WB:	whole body
WBC:	white blood cells
WPB:	whole pelvic bone
Vx:	volume receiving x Gy
WPBM:	whole pelvic bone marrow
